# Direct conversion of methane to formaldehyde and CO on B_2_O_3_ catalysts

**DOI:** 10.1038/s41467-020-19517-y

**Published:** 2020-11-10

**Authors:** Jinshu Tian, Jiangqiao Tan, Zhaoxia Zhang, Peijie Han, Min Yin, Shaolong Wan, Jingdong Lin, Shuai Wang, Yong Wang

**Affiliations:** 1 State Key Laboratory for Physical Chemistry of Solid Surfaces, Collaborative Innovation Center of Chemistry for Energy Materials, National Engineering Laboratory for Green Chemical Productions of Alcohols-Ethers-Esters, and College of Chemistry and Chemical Engineering, Xiamen University, 361005 Xiamen, China; 2 Voiland School of Chemical Engineering and Bioengineering, Washington State University, Pullman, WA 99164 USA

**Keywords:** Heterogeneous catalysis, Natural gas, Chemical engineering

## Abstract

Direct oxidation of methane to value-added C_1_ chemicals (e.g. HCHO and CO) provides a promising way to utilize natural gas sources under relatively mild conditions. Such conversions remain, however, a key selectivity challenge, resulting from the facile formation of undesired fully-oxidized CO_2_. Here we show that B_2_O_3_-based catalysts are selective in the direct conversion of methane to HCHO and CO (~94% selectivity with a HCHO/CO ratio of ~1 at 6% conversion) and highly stable (over 100 hour time-on-stream operation) conducted in a fixed-bed reactor (550 °C, 100 kPa, space velocity 4650 mL g_cat_^−1^ h^−1^). Combined catalyst characterization, kinetic studies, and isotopic labeling experiments unveil that molecular O_2_ bonded to tri-coordinated BO_3_ centers on B_2_O_3_ surfaces acts as a judicious oxidant for methane activation with mitigated CO_2_ formation, even at high O_2_/CH_4_ ratios of the feed. These findings shed light on the great potential of designing innovative catalytic processes for the direct conversion of alkanes to fuels/chemicals.

## Abstract (web summary)

Partial oxidation of methane to value-added C_1_ products remains challenging due to the favorable formation of fully-oxidized CO_2_. Here, the authors show supported B_2_O_3_ catalysts with tri-coordinated BO_3_ units as the active site are highly selective in oxidizing methane to HCHO and CO.

## Introduction

The catalytic transformation of methane to value-added chemicals is of significant interest for the efficient utilization of natural gas sources^1,2^, especially due to the recent shale-gas revolution^3^. In particular, the direct conversion of methane via selective oxidation to C_1_ chemicals (i.e. CH_3_OH, HCHO, and CO) is most attractive, because all of these products are important platform molecules/intermediates for the production of fuels and chemicals^4,5^. Selective oxidation of methane involves the cleavage of C–H bonds and formation of C–O bonds, leading to CH_3_OH, HCHO, and CO as the desired partial oxidation products but also to CO_2_ as an undesired complete oxidation product. Such oxidation processes are induced by nucleophilic attack of active O species at the H or C atom in CH_4_ (or reactive intermediates) before electron transfer^6^. Because the desired C_1_ partial oxidation products (i.e. CH_3_OH, HCHO, and CO) have much higher electrophilicity than CH_4_, they are kinetically more favored in oxidation, leading to dominant formation of undesired CO_2_ at methane conversions even less than 5% on conventional catalysts^7–12^ (e.g., V_2_O_5_^13^, MoO_3_^14^, and Fe_2_O_3_^15^). Lattice O anions exposed on these oxide surfaces act as the nucleophilic and oxidative centers that can be regenerated fast via dissociative adsorption of gaseous O_2_ during catalytic cycles (also described as the Mars van Krevelen mechanism^16^). To our best knowledge, high selectivities to the desired C_1_ partial oxidation products on these traditional metal oxide catalysts are able be obtained merely at low methane conversions (<2%) or low O_2_/CH_4_ ratios (<0.5)^7–15^, making such oxidation processes impractical.

Directly using molecular O_2_ or derived O· radicals to oxidize CH_4_, instead of the more nucleophilic and reactive lattice O anions, would render facile control over the extent of CH_4_ oxidation. Recent studies^17,18^ have shown that nonmetallic B-based materials (e.g. BN^19–21^, B_4_C^22^, SiB_6_^23^, and B_2_O_3_^24,25^) can catalyze oxidative dehydrogenation of C_2_–C_4_ alkanes to alkenes with extraordinarily low selectivities to CO_2_, reflecting, in turn, that these catalysts probably use the moderate O_2_ or O· oxidants to activate the alkane reactants. The BN catalyst has also been attempted for methane oxidation at temperatures above 690 °C, which yielded CO, CO_2_, and methane coupling products (i.e. C_2_H_4_ and C_2_H_6_) with respective selectivities of 76.6, 4.3, and 19.3 at 20.5% methane conversion^26^. We expect that the selectivity of valuable C_1_ products for methane oxidation on the B-based catalysts can be significantly improved at milder temperatures under which HCHO and CH_3_OH can be better stabilized kinetically. Moreover, experiments of nuclear magnetic resonance spectroscopy^27^ and X-ray photoelectron spectroscopy^22^ have revealed that B(OH)_*x*_O_3-*x*_ (where *x* = 0–3) layers formed on the B-based catalysts act as the true active phase, irrespective of their bulk contents.

According to the above considerations, we here study supported B_2_O_3_ catalysts for selective methane oxidation at relatively mild temperatures. Our results show that B_2_O_3_-based catalysts are highly selective in the direct conversion of methane to HCHO and CO, and these selectivities are unexpectedly insensitive to the O_2_/CH_4_ ratios. Structural characterization, kinetic measurements, and isotopic labeling experiments are combined to discern that molecular O_2_ bonded to coordinately unsaturated BO_3_ centers on the B_2_O_3_ surfaces is the crucial oxidant that accounts for the selective methane oxidation. The mechanistic understanding of methane oxidation on the unique B_2_O_3_ surfaces would inspire the design of the next generation of heterogeneous catalysts for selective oxidation of hydrocarbons.

## Results

### Performances of B_2_O_3_-based catalysts in methane oxidation

**Fig. 1:**
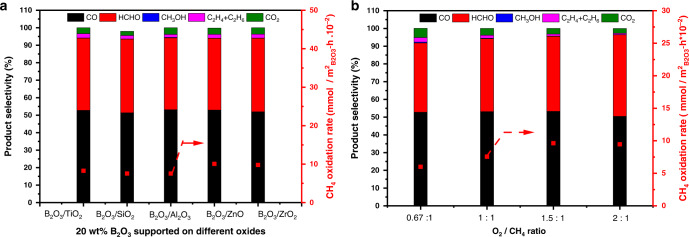
Methane oxidation rates and selectivity on supported B_2_O_3_ catalysts. **a** Effects of oxide support on 20 wt% B_2_O_3_-based catalysts. **b** Effects of O_2_/CH_4_ ratio on 20 wt% B_2_O_3_/Al_2_O_3_. Reaction conditions: 550 °C, 32 kPa *P*_CH4_, 32 kPa *P*_O2_ for (**a**) or 21–64 kPa *P*_O2_ for (**b**), gas composition balanced with N_2_, ~6% CH_4_ conversion was achieved by adjusting the space velocity within a range of 1500–50000 mL g_cat_.^−1^ h^−1^. The methane oxidation rates reported here were normalized by the exposed surface area of B_2_O_3_. The bars in **a** and **b** denote product selectivities (CO in black, HCHO in red, CH_3_OH in blue, C_2_H_4_ and C_2_H_6_ in magenta, and CO_2_ in green), and the red square in **a** and **b** denote methane oxidation rates.

Catalysts containing 20 wt% B_2_O_3_ supported on various oxides (i.e. Al_2_O_3_, SiO_2_, ZnO, TiO_2_, and ZrO_2_) were prepared using the wetness impregnation method with boric acid (H_3_BO_3_) as the boron source (see Methods section). This high B_2_O_3_ loading was chosen to ensure that multiple B_2_O_3_ layers were formed on each oxide support (Supplementary Table 1). Under the conditions studied (O_2_/CH_4_ ratio of 1.0 and 550 °C), these oxide supports themselves were inactive for methane activation, whereas all supported catalysts with 20 wt% B_2_O_3_ selectively converted methane to HCHO and CO (~94 % selectivity with a HCHO/CO ratio of ~1), together with a trace amount of desired CH_3_OH and C_2_ products (C_2_H_4_ and C_2_H_6_), which are irrespective of the nature of the support (Fig. 1a). It is noteworthy that these observed conversions are mainly attributed to the catalytic processes on the B_2_O_3_ surface, instead of gas-phase radical reactions, because the conversion and selectivity of methane oxidation on the supported B_2_O_3_ catalysts had negligible changes whether the empty space of the reactor was fully filled with inert SiC material or not (Supplementary Fig. 1) and their sum did not obey the empirical 100% rule^28^ that was observed in the previous gas phase chemistry (Supplementary Fig. 2).

In all cases, the sum of the selectivities to these desired C_1_ and C_2_ products were above 96%, while the selectivity to undesired CO_2_ was below 4% at ~6% methane conversion (controlled by the space velocity for rigorous selectivity comparison among the examined catalysts). Such high selectivities to the partial oxidation products reflect the superior control of the oxidation extent within the kinetic regime, brought forth by the unique property of the B_2_O_3_-based catalysts as described below, otherwise the fully oxidized CO_2_ would be predominant among the products if the thermodynamic equilibrium is established (e.g., 53.5% methane conversion and 72.8% CO_2_ selectivity at 550 °C and 100 kPa with an initial CH_4_/O_2_ molar ratio of 1/1; Supplementary Fig. 3). This highly selective methane oxidation process could be scaled up by combining efficient separation of the products from the effluent and recycling of the unconverted CH_4_ and O_2_ reactants^29^. Moreover, the fact that the molar ratio of HCHO to CO in the products is ~1.0 makes them potentially desired for the downstream acetic acid synthesis via hydrogenation of HCHO to methanol and its subsequent carbonylation^30,31^ and also for glycolic acid synthesis via direct carbonylation of HCHO^32^. The detected C_2_H_6_ and C_2_H_4_ products are likely ascribed to methane oxidative coupling as we reported previously^21,26^, reflecting the ability of B_2_O_3_-based catalysts to produce C_2+_ molecules from direct methane oxidation. Similar methane oxidation rates (mmol methane converted per surface area of the exposed B_2_O_3_ phase per hour) were obtained for the examined catalysts. These results suggest a negligible effect of the oxide supports on catalytic performance, a result that is consistent with formation of the multiple B_2_O_3_ layers on each oxide support at 20 wt% B_2_O_3_ loading (the loading threshold for forming a B_2_O_3_ monolayer for each of the oxide supports are shown in Supplementary Table 1).

We further studied the effects of O_2_/CH_4_ partial pressure ratios on the performances of the 20 wt% B_2_O_3_/Al_2_O_3_ catalyst at 550 °C, where methane conversions were kept ~6% via adjusting the space velocity for rigorous comparison of reactivity and selectivity. As shown in Fig. 1b, nearly constant selectivities (i.e., >95% selectivity to desired C_1_ and *C*_2_ products with <5% selectivity to undesired CO_2_) were obtained at ~6% methane conversion over a wide range of *P*_O2_/*P*_CH4_ ratios (0.67–2.00), reflective of the unique ability of B_2_O_3_ in preventing complete oxidation of the desired C_1_ and C_2_ products to CO_2_ and thus the potential of high-pressure operation for this catalytic process. Such remarkable selectivities to the desired C_1_ and C_2_ products and a negligible effect of the *P*_O2_/*P*_CH4_ ratio on activity makes this system superior to conventional oxide catalysts (e.g. supported V_2_O_5_ and MoO_3_ in Supplementary Table 2), which must operate at much lower *P*_O2_/*P*_CH4_ ratios (0.1–0.5) to minimize the over-oxidation of C_1_ and C_2_ products by the strongly nucleophilic lattice O anions on metal oxide surfaces^16,33^. Figure 1b also shows that methane oxidation rates increased with increasing the O_2_/CH_4_ ratio, indicating a positive reaction order with respect to O_2_. In addition, these B_2_O_3_-based catalysts exhibited exceptional stability at 550 °C as illustrated over 100 h time-on-stream stable methane oxidation for the B_2_O_3_/Al_2_O_3_ catalyst (Supplementary Fig. 4).

### Active sites of B_2_O_3_-based catalysts for methane oxidation

To provide insight into the nature of active sites on these supported B_2_O_3_ catalysts, ^11^B solid state nuclear magnetic resonance (NMR) was used. Two boron oxide species with chemical shifts centered at 67.9 and 57.1 ppm were observed for these samples (using NaBH_4_ as the reference compound, NMR spectra shown in Supplementary Fig. 5). These are ascribed to tri-coordinated BO_3_ and tetra-coordinated BO_4_ units, respectively^34^. Previous studies^35,36^ suggest that the BO_4_ units are derived from the additional bonding of lattice O anions of the oxide support to the BO_3_ units in B_2_O_3_. The BO_4_/BO_3_ molar ratio of the supported B_2_O_3_ catalysts increased with increasing basic strength of the oxide support (e.g. 0.46 for B_2_O_3_/SiO_2_, 1.45 for B_2_O_3_/Al_2_O_3_, and 1.65 for B_2_O_3_/ZrO_2_ in Supplementary Fig. 5) that can be explained by increased coordination of the B center to more basic lattice O anions of the oxide support. On the other hand, these BO_4_/BO_3_ ratios did not affect the methane oxidation rates of the supported B_2_O_3_ catalysts that were almost identical when normalized by the exposed B_2_O_3_ surface area (Fig. 1a). These results might indicate that the BO_3_ and BO_4_ units have similar catalytic activities.

To further confirm or disapprove our hypothesis, B-substituted ZSM-5 zeolite (B-ZSM-5) was prepared, where the B atoms embedded in the silicate framework were all tetra-coordinated by lattice O anions as confirmed by the single chemical shift of B at 54.1 ppm (Supplementary Fig. S5)^37,38^. Compared with the supported B_2_O_3_ catalysts (e.g., 20 wt% B_2_O_3_/Al_2_O_3_), the B-ZSM-5 sample showed negligible activity in catalyzing methane oxidation with near 45% selectivity to CO_2_ (Supplementary Fig. 6). It is thus concluded that the BO_4_ units with full coordination of B centers are catalytically inactive. The fact that the methane oxidation rates of the supported B_2_O_3_ catalysts are independent of the BO_4_/BO_3_ ratio measured by ^11^B NMR is likely due to that NMR is a bulk technique and that the top layer B_2_O_3_ is not bonded to the oxygen anions of oxide supports on these catalysts with multilayered B_2_O_3_, leading to exclusively active surface BO_3_ units for methane oxidation.

### Mechanism of methane oxidation on B_2_O_3_-based catalysts

Kinetic studies were carried out to provide molecular-level insights into the mechanism of methane activation on supported B_2_O_3_ catalysts. The conversion-selectivity relationship for methane oxidation on 20 wt% B_2_O_3_/Al_2_O_3_ (Supplementary Fig. 7) showed that the selectivities to HCHO and CH_3_OH both monotonically decreased as the conversion of CH_4_ increased from 2 to 10%, concomitant with increased selectivities to CO and CO_2_. These selectivity trends indicate that both HCHO and CH_3_OH are primary products formed from methane conversion. It has been proposed that the initial activation of methane by active O species on oxide surfaces forms bonded methoxy intermediates (CH_3_O) via cleaving a C–H bond in methane, which undergo further dehydrogenation to form HCHO or recombine the cleaved H atom to form CH_3_OH^39,40^. On B_2_O_3_ surfaces, methane selective oxidation to HCHO is the predominant reaction channel for methane activation, as evidenced by the high HCHO/CH_3_OH selectivity ratios obtained at low methane conversions (e.g. ~17 at 2% conversion, Supplementary Fig. 7). The secondary dehydrogenation of HCHO leads to the formation of CO, whereas the CO_2_ product comes from CO oxidation.

**Fig. 2:**
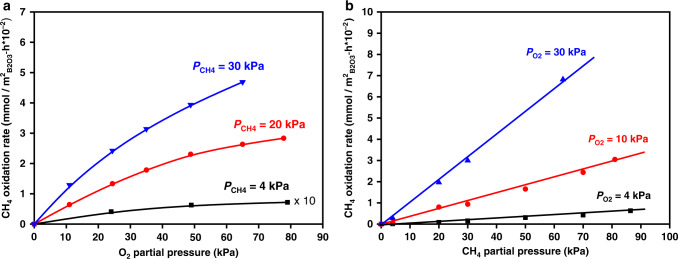
Kinetics of methane oxidation on supported B_2_O_3_ catalysts. Methane oxidation rates as functions of **a** O_2_ pressure and **b** CH_4_ pressure were measured on 20 wt% B_2_O_3_/Al_2_O_3_. Reaction condition: 550 °C, 4–30 kPa *P*_CH4_, 0–80 kPa *P*_O2_ for **a** and 4–30 kPa *P*_O2_, 0–90 kPa *P*_CH4_ for **b**, balanced by N_2_, space velocity at 4920 mL g_cat_.^−1^ h^−1^. In **a**, the rate data measured at CH_4_ partial pressures of 4, 20, and 30 kPa are shown as black square, red cycle, and blue triangle, respectively. In **b**, the rates measured at O_2_ partial pressures of 4, 10, and 30 kPa were shown in black square, red cycle, and blue triangle, respectively. The curves in **a** and lines in **b** represent trends.

Figure 2a depicts that the methane oxidation rate increased with O_2_ pressure (0–80 kPa; 550 °C), but the dependence became weaker at higher O_2_ pressure that is indicative of higher O_2_ coverages on the B_2_O_3_ surface. In contrast, the methane oxidation rate increased linearly with the CH_4_ pressure in the same pressure range, suggesting that CH_4_ barely adsorbs on the BO_3_ sites during catalysis (Fig. 2b), which is consistent with the unfavorable adsorption of CH_4_ on solid surfaces brought forth by its highly symmetrical geometry and weakly polarized C–H bonds^11^. These effects of reactant concentrations on the oxidation rates are indicative of the Eley-Rideal mechanism^41^ on catalytic surfaces, in which one molecule adsorbed on the active site directly reacts with another one from the gas phase. Because of this, we propose that methane oxidation occurs between a gaseous CH_4_ molecule and an O_2_ molecule bonded to the BO_3_ sites. Similar Eley-Rideal-type pathways have been found for methane oxidation on Pd and Pt surfaces at high temperatures^42,43^, which unveils that two adsorbed O atoms formed from the dissociation of a O_2_ molecule on the catalyst surface are required to act concertedly in order to cleave the strong C–H bond of CH_4_.

**Fig. 3:**
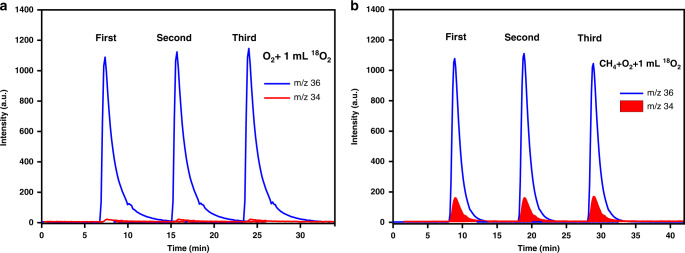
Isotopic assessment of O_2_ activation on supported B_2_O_3_ catalysts. Mass spectra of ^16^O^18^O (*m*/*z* = 34) species upon pulsing ^18^O_2_ (*m*/*z* = 36) into ^16^O_2_ flows on 20 wt% B_2_O_3_/Al_2_O_3_ were collected in the **a** absence and **b** presence of CH_4_ (CH_4_ 1 mL/min, O_2_ 5 mL/min, N_2_ 4 mL/min, temperature: 550 °C, catalyst loading: 0.2 g). In both **a** and **b**, the red and blue curves denote the signals for the m/z ratios of 34 and 36, respectively.

Isotopic labeling experiments were further used to confirm the above hypothesis (i.e. BO_3_-surface-bonded O_2_ directly activates CH_4_). Pulses of a small amount of ^18^O_2_ into flowing ^16^O_2_ on the supported B_2_O_3_ catalysts at 550 °C did not lead to the formation of isotope-exchanged ^18^O^16^O species, excluding the presence of dissociative adsorption of O_2_ molecules on the B_2_O_3_ surface (Fig. 3a). In contrast, when CH_4_ was co-fed with ^16^O_2_ and pulses of ^18^O_2_ over the surface of the same B_2_O_3_ catalysts, a significant amount of ^18^O^16^O was detected (Fig. 3b). We thus infer that the O–O bond in the adsorbed O_2_ molecule is cleaved concertedly when it activates the C–H bond of CH_4_.

**Fig. 4:**
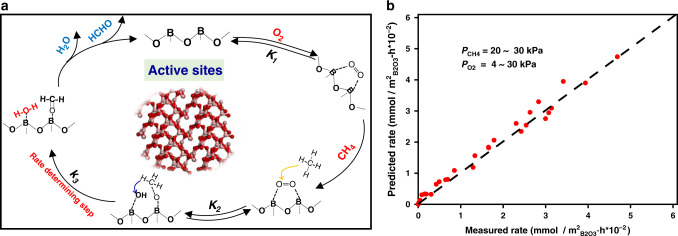
Mechanistic insights for methane activation on a B_2_O_3_ surface. **a** Schematic diagram of the plausible pathway of methane selective oxidation to formaldehyde on B_2_O_3_-based catalysts. **b** Parity plots for the measured rate data of methane selective oxidation on B_2_O_3_/Al_2_O_3_ and those predicted using Eq. 1 (the regression-fitted parameters shown in Supplementary Table 3). In **a**, *K*_1_ and *K*_2_ are equilibrium constants for the corresponding steps, and *k*_3_ is the kinetic constant for hydrogen abstraction of the surface methoxy species by a neighboring hydroxyl.

The above kinetic and isotopic assessments are combined to give a plausible pathway for the formation of HCHO from CH_4_ oxidation on B_2_O_3_-based catalysts as described in Fig. 4a. First, O_2_ adsorbs on two vicinal BO_3_ units with each O atom in O_2_ bound to one of the electron-deficient B centers (Step 1, Fig. 4a). A gaseous CH_4_ molecule then attacks this adsorbed O_2_ reactant, resulting in concurrent formations of hydroxy and methoxy species (Step 2, Fig. 4a). Formaldehyde and H_2_O are further produced from hydrogen abstraction of the methoxy moiety by a hydroxyl (Step 3, Fig. 4a). Desorption of these products from the active BO_3_ sites completes a catalytic turnover for methane partial oxidation on B_2_O_3_ (Step 4, Fig. 3a). The measurable O-exchange between O_2_ molecules in the presence of CH_4_ (Fig. 3b) indicates the reversibility of Steps 1 and 2 on B_2_O_3_ under reaction conditions. This suggests, in turn, that Step 3 is kinetically relevant. This finding is consistent with a previous report^6^ that the electronegativity of oxide catalysts affects the selectivity to HCHO. These elementary steps, taken together with the pseudo-steady-state approximation for all bound species and the quasi-equilibrated nature of all steps except Step 3, lead to an equation for methane conversion rates (*r*):

1 $$r = \frac{{k_3K_1K_2P_{{\mathrm{O}}2}P_{{\mathrm{CH}}4}}}{{1 + K_1P_{{\mathrm{O}}2}}}$$

Here, *K*_1_ and *K*_2_ are the respective equilibrium constants for Steps 1 and 2, whereas *k*_3_ is the kinetic constant for Step 3. The functional form of Equation 1 accurately describes all methane oxidation rate data measured within a wide reactant pressure range (Fig. 4b; regression-fitted parameters shown in Supplementary Table 3), supporting the proposed methane activation mechanism on the B_2_O_3_ surfaces (Fig. 4a).

## Discussion

In summary, nonmetallic B_2_O_3_-based catalysts are selective and stable in the partial oxidation of methane to HCHO and CO. Surface tri-coordinated BO_3_ units are the active sites for methane oxidation. O_2_ molecules bound to the electron-deficient B centers of these BO_3_ units are moderate oxidants for methane activation, exhibiting strong suppression of the formation of thermodynamically favored CO_2_. Further exploitation of such nonmetallic oxide catalysts will bring innovative strategies and catalyst systems for efficient and selective oxidation of methane (and other alkanes) to valuable chemicals.

## Methods

### Preparation of B_2_O_3_-based catalysts

B_2_O_3_ catalysts supported on various oxides (including Al_2_O_3,_ TiO_2_, ZnO, ZrO_2_, and SiO_2_) were prepared by wetness impregnation method using boric acid (Sinopharm chemical reagent co. LTD) as the boron source. The preparation method was described below using B_2_O_3_/Al_2_O_3_ as an illustrative example: a certain amount of H_3_BO_3_ was dissolved in deionized water (10 mL) and then the resulting aqueous solution was added dropwise dropped to pure Al_2_O_3_ (0.5 g). After stirring vigorously for 1 h, the impregnated samples were heated to 65 °C and subsequently vacuumed at 65 °C for 8 h. The as-prepared products were then calcined at 600 °C for 5 h in air.

### Preparation of B-ZSM-5 samples

B-ZSM-5 was synthesized according to a previously reported method^37^. Typically, NaOH, 1–6 hexanediamine (HMDA) and tetrapropylammonium bromide (TPABr) were dissolved in 18.9 mL of deionized water. The solution was stirred for 30 min, and then 1.8 g of porous SiO_2_ (Aladdin) and 0.3373 g H_3_BO_3_ were slowly added. The gel was stirred for 1 h and then transferred into a teflon-lined stainless-steel autoclave for crystallization at 180 °C for 48 h. The obtained samples were separated by filtration, washed with deionized water, dried at 90 °C for 12 h and finally calcined under static air at 550 °C for 5 h.

### Measurement of catalytic performance

Catalytic methane oxidation was conducted using a fixed-bed quartz tubular reactor (7 mm inner dimeter) with plug-flow hydrodynamics. The B_2_O_3_-based catalyst (0.15–0.18 mm sieved particles, ~200 mg, corresponding to a volume of 0.38 mL) was first pretreated in flowing O_2_/N_2_ (1/1 in volume) for 2 h under 580 °C and then cooled to the reaction temperature under N_2_. CH_4_/He (90/10%), O_2_ (99.99%), and N_2_ (99.99%) were individually controlled using three mass flow controllers (Sevenstar Technology Co., Ltd) to provide the reaction gas feed. The feed rate reported here is the weight hourly space velocity (WHSV), in which the gas volume refers to the standard ambient temperature and pressure. The concentrations of reaction products in the effluent were analyzed by an online gas chromatography (GC2060, Shanghai Ruimin GC Instruments, Inc). Samples in the quantitative ring were separated by Porapak column (6 m×3 mm) and then quantified using a thermal conductivity detector (TCD) for He, CH_4_, and CO_2_^44^. The other gases are introduced into a flame ionization detector (FID) and subsequently analyzed including CO, CH_4_, CO_2_, C_2_H_4_, C_2_H_6_, HCHO, and CH_3_OH. Control experiments with SiC showed that there was negligible methane conversion without the catalyst. In all tests, carbon mass balances exceeded 98%. The CH_4_ conversion (*X*_CH4_) and the carbon selectivity of each product *i* (*S*_i_) were calculated using a standard normalization method (He as internal standard gas) based on the carbon balance, which were defined as

2 $$X_{{\mathrm{CH}}4} = \left( {{\mathrm{1}} - \frac{{P_{{\mathrm{CH}}4}^{{\mathrm{out}}}}}{{P_{{\mathrm{CH}}4}^{{\mathrm{in}}}}}} \right) \times 100\%$$

3 $$S_i = \frac{{n_iP_{\mathrm{i}}^{{\mathrm{out}}}}}{{{\sum} {n_i} P_{\mathrm{i}}^{{\mathrm{out}}}}} \times 100\%$$

Here, $P_{{\mathrm{CH}}4}^{{\mathrm{out}}}$ and $P_{{\mathrm{CH}}4}^{{\mathrm{in}}}$ are the corresponding partial pressures of methane at the outlet and inlet of the reactor, while $P_{\mathrm{i}}^{{\mathrm{out}}}$ and *n*_*i*_ are the outlet partial pressure and the carbon number of each product *i* formed from methane oxidation, respectively.

### Structural characterization

Specific surface areas of catalysts were measured by the Brunauer–Emmett–Teller (BET) method, using a Micromeritics Tristar 3020 surface area and porosimetry analyzer. Prior to measurement, all samples were degassed at 150 °C for 6 h. ^11^B solid nuclear magnetic resonance (^11^B-NMR) analysis was recorded on a Bruker NMR 500 DRX spectrometer at 500 MHz and referenced to NaBH_4_ (3.2 ppm). Isotopic labeling experiments were performed in a fixed-bed single-pass flow micro-reactor. A mixture of N_2_ and ^16^O_2_ (research grade, 99.99%) was fed to the 20 wt% B_2_O_3_/Al_2_O_3_ catalyst bed at 550 °C until the baseline was stabilized and then an ^18^O_2_ (Cambridge Isotope Lab., 99%; 1 mL each time) pulse was injected into the flow using a syringe. The chemical and isotopic compositions of the reactor effluent were measured by online mass spectrometry (MS, Pfeiffer, OminStar TM) at intervals of 10 s with a m/z scanning from 1 to 50. The m/z signals of 32, 34, and 36 represent ^16^O^16^O, ^16^O^18^O, and ^18^O^18^O, respectively.

## Supplementary information

Supplementary Information

Peer Review File

## Data Availability

The datasets generated and analyzed during the current study are available from the corresponding authors upon a reasonable request.
