# Supplementary material for: Direct conversion of methane to formaldehyde and CO on B2O3 catalysts
Source: Nat Commun. 2020 Nov 10;11:5693. doi: 10.1038/s41467-020-19517-y (PMC7655938; doi:10.1038/s41467-020-19517-y)
Supplement: Supplementary file 1 — Supplementary Information [file 41467_2020_19517_MOESM1_ESM.docx]

**Supporting Information**

**Direct Conversion of Methane to Formaldehyde and CO on B_2_O_3_ Catalysts**

**Jinshu Tian^1,†^, Jiangqiao Tan^1,†^, Zhaoxia Zhang^1,†^, Peijie Han^1^, Min Yin^1^, Shaolong Wan^1^, Jingdong Lin^1^, Shuai Wang^1*^, Yong Wang^2 *^**

^1^ State Key Laboratory for Physical Chemistry of Solid Surfaces, Collaborative Innovation Center of Chemistry for Energy Materials, National Engineering Laboratory for Green Chemical Productions of Alcohols-Ethers-Esters, and College of Chemistry and Chemical Engineering, Xiamen University, Xiamen 361005, China.

^2^ Voiland School of Chemical Engineering and Bioengineering, Washington State University, Pullman, WA 99164, USA.

^*^ Corresponding authors: [shuaiwang@xmu.edu.cn](mailto:shuaiwang@xmu.edu.cn) (Shu.W.); [yongwang@pnnl.gov](mailto:yongwang@pnnl.gov) (Y.W.)

^†^ These authors contributed equally to this work.

**Supplementary Fig. 1.** The comparison of catalytic performance for methane oxidation reaction on 20 wt% B_2_O_3_/Al_2_O_3_ with and without filling the empty space of the reactor. Reaction conditions: 550ºC, 32 kPa *P*_CH4_, 32 kPa *P*_O2_, balanced with N_2_, inert SiC was used to fill the empty space.

**Supplementary Fig. 2.** The sum of the CH_4_ conversion and selectivities to the partial oxidation products and the corresponding carbon balance as a function of CH_4_ conversion for the 20 wt% B_2_O_3_/Al_2_O_3_ catalyst. Reaction conditions: 550ºC, 32 kPa *P*_CH4_, 32 kPa *P*_O2_, gas composition balanced with N_2_, the CH_4_ conversion was varied by adjusting the space velocity within a range of 4000-50000 mL g_cat_^-1^ h^-1^.


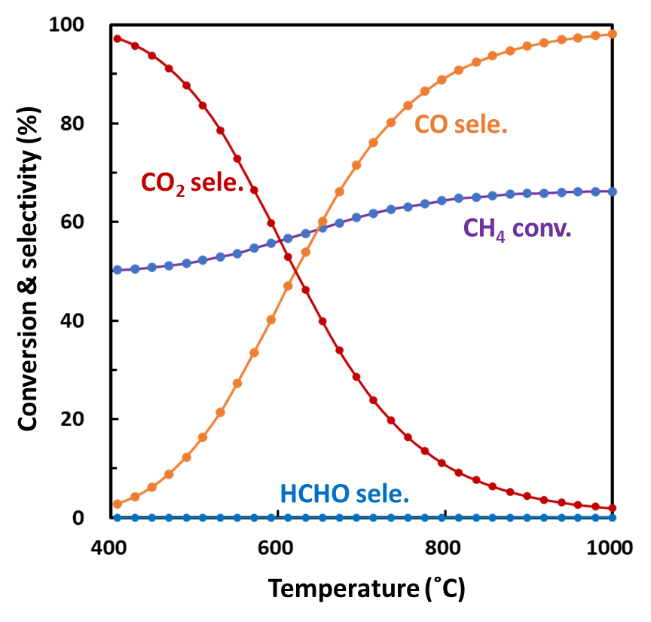


**Supplementary Fig. 3.** Calculated equilibrium conversion of CH_4_ and carbon selectivities of HCHO, CO, and CO_2_ among the oxidation products for a mixture of CH_4_ and O_2_ as functions of temperature (initial CH_4_/O_2_ molar ratio = 1/1, 100 kPa).

**Supplementary Fig. 4.** Time-on-stream performance of the 20 wt% B_2_O_3_/Al_2_O_3_ catalyst for methane oxidation. Reaction condition: 550˚C, *P*_O2_ = *P*_CH4_ = 32 kPa, N_2_ as the balance gas, WHSV = 4650 mL/g_cat_-h, ~6% CH_4_ conversion.

**Supplementary Fig. 5.** ^11^B nuclear magnetic resonance spectra of B_2_O_3_ catalysts supported on various oxides (Al_2_O_3_, ZrO_2_, and SiO_2_) and B-substituted ZSM-5 zeolites (B-ZSM-5).

**Supplementary Fig. 6.** Catalytic performance of B-ZSM-5 and 20 wt% B_2_O_3_/Al_2_O_3_ catalysts in methane oxidation. Reaction condition: 550˚C, P_O2_ = P_CH4_ = 32 kPa, N_2_ used as the balance gas, WHSV = 4650 mL/g_cat_-h.

**Supplementary Fig. 7.** Product selectivities as a function of methane conversion on the 20 wt% B_2_O_3_/Al_2_O_3_ catalyst. Reaction condition: 550˚C, *P*_CH4_= *P*_O2_ = 32 kPa, N_2_ used as the balance gas, WHSV= 2325-11625 mL/g_cat_-h.

**Supplementary Table 1.** Special surface area of studied oxides and theoretical loading values for forming a B_2_O_3_ monolayer on these oxide support surfaces.

| Supports | S_BET_ (m^2^/g) | Theoretical loading for  forming a B_2_O_3_ surface monolayer (%) |
| --- | --- | --- |
| Al_2_O_3_ | 162 | 12 |
| ZnO | 4 | 0.5 |
| ZrO_2_ | 17 | 1.5 |
| TiO_2_ | 49 | 3.5 |
| SiO_2_ | 160 | 10 |

**Supplementary Table 2.** Comparison of activity and selectivity among typical solid catalysts for partial oxidation of methane.

| Catalysts | Temp. (˚C) | P_CH4_ (kPa) | O_2_/CH_4_  ratio | CH_4_  conversion (%) | Carbon selectivity (%) | | | | Reaction rate (mmol_CH4_ g_cat_^-1^ h^-1^) | Turnover  frequency (h^-1^) | Ref. |
| --- | --- | --- | --- | --- | --- | --- | --- | --- | --- | --- | --- |
|  |  |  |  |  | HCHO | CH_3_OH | CO | CO_2_ |  |  |  |
| MoO_3_/SiO_2_ | 550 | 16.8 | 0.44 | 1.2 | 12.0 | 1.0 | 35.0 | 51.0 | -- | -- | a |
| VO_x_/SBA-15 | 600 | 20.2 | 0.13 | 1.8 | 36.4 | 0.8 | 58.7 | 4.5 | 97 | 126^k^ | b |
| CuO_x_/SBA-15 | 625 | 33.8 | 1.00 | 2.8 | 44.0 | -- | 28.0 | 28.0 | 17.5 | -- | c |
| FePO_4_/SBA-15 | 650 | 33.8 | 0.50 | 3.3 | 36.0 | -- | -- | -- | 7.3 | 21.9^k^ | d |
| VO_x_/SiO_2_ | 650 | -- | 0.11 | 4.5 | 32.0 | -- | 57.0 | 11.0 | 88 | 220^k^ | e |
| VO_x_/MCM-41 | 650 | 53.8 | 0.13 | 5.4 | 22.0 | 0.2 | -- | -- | 210 | 374^k^ | f |
| CsPW_11_CoO_39_ | 650 | 72.1 | 0.40 | 6.0 | 22.5 | 6.6 | 34.9 | 36.0 | -- | -- | g |
| WO_x_/SiO_2_ | 650 | 84.9 | 0.19 | 6.9 | 11.9 | -- | 25.1 | 63.0 | 7.8 | 12.0^k^ | h |
| MgO-B_2_O_3_/SiO_2_ | 600 | 38.0 | 0.33 | 3.5 | 18.6 | -- | -- | -- | 1.3 | 0.7^k^ | i |
| MoO_3_-SiO_2_ | 620 | 90.0 | 0.11 | ~4.8 | ~27 |  | ~48 | ~14 | -- | -- | j |
| B_2_O_3_/Al_2_O_3_ | 550 | 32.0 | 1.0 | 5.9 | 41.1 | 0.3 | 53.1 | 3.8 | 9.0 | 16.8^k^ | this work |
| B_2_O_3_/Al_2_O_2_ | 550 | 32.0 | 2.0 | 6.8 | 46.0 | 0.1 | 50.4 | 2.7 | 10.4 | 19.4^k^ | this work |

(a) *Catal. Today*, 1998, 45, 29-33; (b) *Appl. Catal. A*, 2003, 249, 345-354; (c) *J. Phys. Chem. C*, 2008, 112, 13700-13708; (d) *Catal. Today*, 2004, 93, 155-161; (e) *Top. Catal.*, 2017, 60, 1129-1139; (f) *J. Catal.* 2000, 191, 384-400; (g) *J. Mol. Catal. A*, 2013, 379, 255-262; (h) *Appl. Catal. A*, 1999, 184,143-152; (i) *J. Catal.*, 1987, 108, 252-255; (j): *AIChE J.*, 1987, 33, 1808-1812; (k) estimated assuming that all the sites exposed on the catalyst are active for methane oxidation.

**Supplementary Table 3.** The regression-fitted parameters for the functional form of Equation 1 in the main text.

| Catalysts | $k_{3}K_{1}K_{2}$  (nmol/g_cat_-h-kPa^2^×10^-2^) | $K_{2}$  (kPa^-1^×10^-4^) | Coefficient of association (R) |
| --- | --- | --- | --- |
| 20 wt% B_2_O_3_/Al_2_O_3_ | 185 ± 2 | 132 ± 2 | 99.9% |
